# Acceptability of a cessation intervention for pregnant smokers: a qualitative study guided by Normalization Process Theory

**DOI:** 10.1186/s12889-020-09608-2

**Published:** 2020-10-06

**Authors:** Susan E. Jones, Sharon Hamilton, Ruth Bell, Vera Araújo-Soares, Martin White

**Affiliations:** 1 School of Health and Life Sciences, Teesside University, Borough Road, Middlesbrough, TS1 3BX UK; 2 Institute of Health and Society, Newcastle University, Newcastle upon Tyne, UK; 3 Department of Health Technology and Services Research, University of Twente, Enschede, Netherlands; 4 MRC Epidemiology Unit, School of Clinical Medicine, University of Cambridge, Cambridge, UK

**Keywords:** Complex intervention, Normalization Process Theory, Process evaluation, Qualitative, Smoking cessation, Pregnancy, Opt out, Stop smoking services

## Abstract

**Background:**

Smoking during pregnancy has serious consequences for maternal and child health. An intervention package to embed National Institute for Health and Care Excellence guidance (babyClear©) was delivered across maternity and stop smoking services (SSS) within an English region, to support pregnant women to stop smoking. We aimed to ascertain acceptability among pregnant smokers receiving the intervention.

**Methods:**

Pregnant smokers who received the intervention and participated in the study were interviewed, first at around 16 weeks of pregnancy (*n* = 17) and again several weeks later (*n* = 8) or postpartum (*n* = 3). Interview schedules were informed by Normalization Process Theory (NPT) and Theoretical Domains Framework; interviews were audio-recorded, transcribed and analysed thematically, using the Framework method and NPT. Findings are grouped according to the four NPT concepts.

**Results:**

Coherence: Carbon monoxide monitoring appeared to make sense; women were motivated to quit by being monitored. Cognitive participation: When linked to a professional discourse of caring and concern, some women were prompted to engage with the SS message. Women were more guarded in their reaction to initial contact from the SSS; reporting attending appointments successfully, or in some cases, experiencing problems that decreased engagement and made quitting harder. Collective action: Where women continued to smoke or failed to attend SSS appointments, an extra intervention was delivered, the Risk Perception Tool (RPT), which often prompted pregnant women to act. Reflexive monitoring: Most women accepted the need for a hard-hitting approach (RPT) and, while it distressed them at the time, they claimed they were subsequently grateful for it. SSS intervention post-RPT was seen as supportive, partly because it often involved home visits. Aspects of family inclusion in babyClear© were reported as beneficial. In Trusts where women experienced services as less focused on prioritising the stop smoking message, less well integrated or reported maternity staff as less adept at delivering the RPT, women found babyClear© less acceptable overall.

**Conclusions:**

The babyClear© package was acceptable to pregnant smokers interviewed during and shortly after pregnancy and, in some cases, to promote quitting. However, some contexts were more optimal than others, leading to variation in acceptability overall.

## Background

Smoking in pregnancy is associated with increased risk of serious adverse pregnancy outcomes such as miscarriage and stillbirth [1, 2], intrauterine growth restriction and low birth weight [3, 4]. In addition, there are other short and long-term health consequences for children born to mothers who smoke [5, 6] and significant annual costs to the National Health Service (NHS) for treating these mothers and their children [7].

A meta-synthesis of qualitative research on women who commence pregnancy as smokers has identified several reasons why some women struggle to quit [8]. Smoking in pregnancy is strongly socially patterned; women living in disadvantaged circumstances are more likely to smoke prior to pregnancy, and to find it harder to quit while pregnant or maintain a quit attempt postpartum [9]. This is due to the embeddedness of smoking in these women’s lives and how it shapes their social identities, making it more likely that cessation attempts during pregnancy will be seen as only temporary changes [8, 10]. Those who are successful are more likely to have support in quitting from close friends or relatives [8, 11, 12].

The North East has the highest rates of smoking in pregnancy in England [13]. Shortly before this study began in 2012, rates of smoking amongst pregnant women were in excess of 20% [13]. Despite the introduction of National Institute for Health and Care Excellence (NICE) public health guidance 26, ‘*How to stop smoking in pregnancy and following childbirth*’ [14], midwifery services in the region had generally been slow to implement change [15–17].

### The intervention

In August 2012, Fresh (the North East Tobacco Control Office) commissioned the Tobacco Control Collaborating Centre (TCCC), now part of Improving Performance in Practice (IPiP), to develop and deliver a comprehensive package of support known as ‘babyClear©’ to transform services so that they effectively supported pregnant smokers to stop smoking. The key elements of the babyClear© package, which had been developed and tested by the TCCC, are listed. The intervention is based on the evidence and recommendations of NICE guidance [14] and evidence (including a study by Beenstock et al. [15]) that aimed to understand barriers and facilitators associated with implementation of the guidance.

#### Key elements of the babyClear© package

1. A protocol driven referral pathway, based on universal carbon monoxide (CO) monitoring at booking by midwives as part of routine care, which specifies thresholds for referral and actions to be taken
2. Training of all midwives both in the use of CO monitors, thresholds and systems for referral, and also in delivering a brief intervention to encourage engagement with stop smoking services (SSS)
3. Training of SSS advisors in giving effective advice to pregnant smokers
4. Training of SSS administrative staff in effective customer relationship management techniques, in order to convert more referrals into appointments
5. Additional contact from SSS at specified frequencies
6. An intensive Risk Perception Tool (RPT), delivered by trained midwives following the dating ultrasound scan to smokers who have not engaged with SSS
7. Branded materials and equipment, including CO monitors, to support the referral pathway, and training in stop smoking interventions for pregnant women
8. Information systems to capture data on the implementation and delivery of the intervention package.

Thus, pregnant smokers would experience universal screening rather than discretionary selection by midwives, opt out (rather than opt in) referral into Stop Smoking Services (SSS) and higher levels of ongoing support, than had been previous practice. For those still smoking at the 12-week dating ultrasound scan, an additional intervention, the Risk Perception Tool (RPT) was delivered. During the RPT mother’s and baby’s carbon monoxide (CO) readings were displayed on a computer screen with the image of a baby changing from green, through amber to red and flashing, if readings were high. This RPT session also involved a midwife using a fetal doll to demonstrate the direct effect of smoking on the baby. On occasion, partners or relatives were present. For further details of the intervention see Bell at al. [16].

The overall aim of the evaluation, of which this study formed a part, was to determine whether a complex service reconfiguration improved the delivery of smoking cessation interventions to pregnant smokers, whether the reconfigured service could be implemented and sustained effectively, whether it cost-effectively resulted in improved pregnancy outcomes and whether it was acceptable to both health professionals and pregnant smokers. The effect of the intervention package on referral rates to SSS, quit rates and pregnancy outcomes (low birthweight for gestational age and preterm delivery), as well as the cost-effectiveness of the intervention (NHS costs per additional quit), is reported elsewhere [16], as is staff views of intervention implementation and prospects for normalization in midwifery and stop smoking services [17].

The aim of the research reported in this paper was to assess the acceptability of reconfigured services among pregnant women who were offered stop smoking support as a result of the system-wide babyClear© intervention package [18]. Smoking cessation interventions during pregnancy can be effective in reducing smoking into late pregnancy [19]. This study adds to the evidence base by analysing accounts from pregnant smokers who experienced the intervention package, involving several activities designed to overcome some of the existing barriers to women accessing and receiving continued support from SSS, such as inconvenience, delay and low prioritisation of stop smoking. The driving question for this element of the evaluation study was: to what extent did pregnant women who smoked find these intervention activities acceptable?

## Methods

The study design aimed to elicit the perceptions of pregnant women who had experienced the complete babyClear© package. However, the implementation of the new package of care across all hospital Trusts and local authority departments in the region was slower than anticipated [17]. Roll out of the risk perception element was not always complete at the time of data gathering, so only the four Trust areas where full implementation had taken place were selected. For details of local contexts that influenced uptake of the intervention please refer to Jones et al. [17].

### Sample

Maternity and SSS staff handed invitations to the women, allowing them to make contact with the researcher if willing to participate. In total, more than 185 invitation sheets were handed out by staff to women who had received the RPT, using an agreed script, whenever they had opportunity, including mention of financial compensation: a £50 high street voucher, to be given only on completion of two interviews. All pregnant women who responded to the invitation (*n* = 17) were sent an information sheet and agreed to be interviewed; their ages varied between 18 and 39 years (3 in their teens, 9 in their 20s and 5 in their 30s). Two were married, 15 had a partner, out of which 8 cohabited. All were of white British origin. All were smoking at conception, 9 (out of 17) were smoking at first interview; 11 participated in second interviews of whom 4 remained quit, 2 more quit and 5 remained smoking.

### Data collection

Interviews took place first at around 16 weeks of pregnancy and again prenatally (*n* = 8; 5–10 weeks between interviews) or postpartum (*n* = 3; 26–34 weeks between interviews), as determined by the time of recruitment and the end of the data collection period. The majority of the women were interviewed by SJ, who has clinical nursing and public health research experience, either at home or in community settings or by telephone, whichever was most convenient for the participant (Table 1).

**Table 1:** Setting for data collection

Method	Interview 1	Interview 2
Face-to-face	13^a^	4
Telephone	4	7
Neither	0	6^b^
INTERVIEW TOTAL	17	11

^a^Husband present in one home interview

^b^Five declined a second interview and one was recruited too late within the data collection cycle

A semi-structured interview schedule (Supplementary information 1) was developed in collaboration with our service user reference panel, covering CO monitoring, behaviour change, SSS, relationship with midwife and personal views/attitudes. Two frameworks of understanding underpinned questioning: Normalization Process Theory (NPT) [21] (Table 2), and the Theoretical Domains Framework (TDF) [22, 23]. Researchers used NPT to assess potential for normalization of the intervention in the study as a whole, and thought it would be interesting to use it to assess how pregnant women’s perceptions on acceptability affected normalization too. This was of interest because NPT is more commonly used with staff data. Health professionals’ understanding and implementation of the intervention are reported elsewhere [17]. The TDF draws together different theories - used to explain individual behaviour change - into one, cohesive format, making it useful in prompting interviewees to think about their feelings and choices associated with this intervention [22, 23]. Results from data analysis using the TDF will be reported elsewhere.

**Table 2:** Normalization Process Theory constructs

Construct	Definition
Coherence	The process of *sense-making* and *understanding* that individuals and organisations have to go through in order to promote or inhibit the routine embedding of a practice to its users. These processes are energized by *investments of meaning* made by participants.
Cognitive Participation	The process that individuals and organisations have to go through in order to *enrol* individuals to *engage* with the new practice. These processes are energized by *investments of commitment* made by participants.
Collective Action	The work that individuals and organisations have to do to *enact* the new practice. These processes are energized by *investments of effort* made by participants.
Reflexive Monitoring	The *informal and formal appraisal* of a new practice once it is in use, in order to assess its advantages and disadvantages and which develops users’ comprehension of the effects of a practice. These processes are energized by *investments in appraisal* made by participants.

Taken from Finch et al. [20]

First interviews lasted between 18 and 63 min (average 28), and second interviews lasted between 8 and 20 min (average 12). Telephone interviews tended to be shorter than face-to-face conversations; some women were inclined to talk at length, while others kept their comments to a minimum, although the reasons for these differences were not explored. Field notes were written up following data collection and throughout the study. Data saturation was not reached due to delayed roll out of the intervention and slow recruitment of participants. Interviews were digitally recorded with permission and subsequently transcribed verbatim by professional transcribers.

### Data analysis

**Fig. 1:**
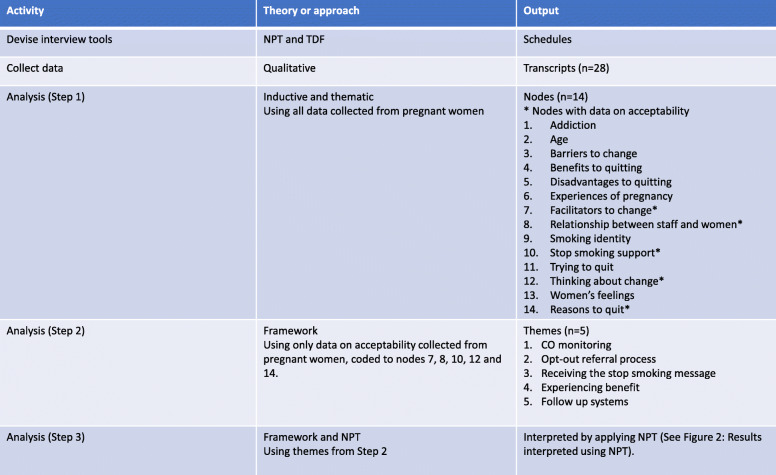
Analytical process

NVivo 10 software was used for data management. The data from pregnant women were inductively and thematically analysed (SJ) and nodes (*n* = 14) agreed through ongoing discussion (between SJ, JS and SH). Five nodes contained data relating to acceptability. Data in other nodes were excluded from this analysis as the research question focused on acceptability. These data were extracted, indexed and themed using a framework approach [24]. The data were then summarised, tabulated and used to create charts for each theme; then grouped to interpret the data as a whole, using NPT concepts. This rearrangement of the data under themes associated with acceptability to pregnant women is the basis for this paper (Fig. 1).

### Public and patient user involvement

In addition to the fieldwork as described above, the service user reference panel, which consisted of two groups, with three smokers in each, who were also mothers of young children, were convened to advise on such matters as the design of the research instruments, methods of recruitment and interpretation of the findings. Their input focused on increasing the effectiveness and accuracy of the evaluation in two main ways: by shaping service user recruitment methods and the language and content of interview schedules to serve as data collection methods with staff and service users. Working with the panel also increased researcher awareness of the issues from a public and patient perspective.

## Results

**Fig. 2:**
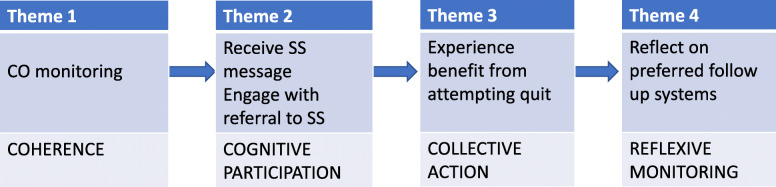
Results interpreted using Normalization Process Theory

Five main themes relating to acceptability of the intervention emerged, these were linked to NPT concepts: CO monitoring (Coherence); opt out referral process, receiving the stop smoking message (Cognitive Participation); experiencing benefit (Collective Action), and; follow up systems (Reflexive Monitoring) (Fig. 2). Quotes were chosen during Framework Analysis according to those which encapsulated the concept or idea or view most aptly. Those included in this paper were deliberately chosen from multiple participants.

### Carbon monoxide monitoring

CO monitoring made sense (coherence) to smokers i.e. it was accepted, even expected after the first visit.

*Interviewer: So what did you think about it when they first asked you to blow into the monitor?*

*PW: I was a bit sceptical at first cos I was thinking ‘God, what’s it gonna ... you know show up’, and then when it did show up it was like it hit home basically, to say you are not just doing damage to yourself, you are doing it to the baby, and it is just like, God, you don’t realise how much carbon monoxide you do actually intake.*

Pregnant woman (PW52), Interview 1

It was expected by some women that CO readings would be taken at every maternity appointment, as well as any SSS appointments, and this was part of the intervention protocol. However, this did not always occur, variation in when and how frequently CO monitoring took place was reported by women. They said that sometimes smoking was discussed by midwives but without CO monitoring.

*Interviewer: So did the midwife mention it [smoking] at your different appointments?*

*PW: No, I don’t think she has really. I think they did ask us, like have we still stopped, and when I says, aye, she said, that’s great.*

*Interviewer: Did they ever check your carbon monoxide again with the monitor?*

*PW: No, they didn’t.*

*Interviewer: Just left it really?*

*PW: Mmm.*

Pregnant woman (PW68), Interview 2

However, monitoring was consistently reported at the RPT, in follow-up visits by care assistants or the public health midwife and in SSS clinics.

### Opt out referral process

Women engaged with the idea (cognitive participation) that midwife appointments at various stages in pregnancy (i.e. early bird, pre-6-8 week’s gestation; booking-in, usually 6–8 weeks gestation; RPT, 10–12 weeks gestation) or subsequent appointments, were used as opportunities to refer them to the SSS. Women reported that some midwives spent more time discussing smoking than others.

With regard to initial contact from the SSS, one woman reported attending appointments successfully:

*I was only about nine weeks, I wasn’t very far along at all, but I think actually at the time I last spoke to you I wasn’t very keen on getting back in touch with the stop smoking services anyway, but with the help of the midwife as well, now I have had quite a few appointments with her, I did get back in touch with her [stop smoking advisor].*

Pregnant woman (PW620), Interview 2

Another woman, where the opt out referral service model to the SSS included home visits from a midwife, identified less flexibility over appointment times and reported this made quitting harder.

*(Partner of PW): And they make appointments at like three o'clock and that [school pick-up time].*

*PW: And six weeks [school] holidays … Because she'd come ... I said what day I was getting married and she wanted to come I think it was five o'clock the night before. I said you've got no chance, I'm sorry but no.*

Pregnant woman (PW71), Interview 1

### Receiving the stop smoking message

The content of the babyClear© smoking cessation programme was broadly acceptable, even welcomed. Most women accepted the need for a hard-hitting approach, that pointedly targeted and warned of the dangers of smoking to their baby, an approach one participant described as “quite distressing”, but they were grateful for it, saying: “it really hit me [when using the doll and placenta]” (PW 534, Interview 1), “it makes you think about it a lot” (PW 547, Interview 1).

*I don’t know, the way they got the information over, and the way they did it was what I needed, was the shock I needed to actually do it. If they’d done it softer with us I wouldn’t … . kind of would have just brushed it off.*

Pregnant woman (PW66), Interview 1

If the smoking cessation goal was seen as unachievable, inconsistent or still contradicted their personal worldview, it was perceived as less acceptable. Where women experienced services as well integrated, with established feedback loops, they reported feeling some benefit; but where services appeared to them to be less focused on prioritising the stop smoking message, disjointed or less adept at delivering the RPT, women found babyClear© less acceptable.

*… Yes, I am all over different hospitals because of different certain things, but no, I don’t get really feedback [about my attempts to quit smoking]. I get told off I think more when I go, when they check me with the midwife and the doctor.*

Pregnant woman (PW52), Interview 1

### Experiencing benefit

Women were motivated to act i.e. to quit (collective action), by CO monitoring and the RPT; although there was a sense of conflict.

*I welcome it [the RPT] because it does scare you into ‘you need to stop’. But then, on the other hand, I know everyone is entitled to do their own thing, from the point of view they can have their own opinions. So it kind of pressurises people into, you have to stop smoking otherwise your breathing will be damaged or your breathing will have problems.*

Pregnant woman (PW715), Interview 1

Women reported taking action when the intervention was linked to a professional discourse of caring and concern.

*Well, she [the midwife] asked me if I smoked and I said yes, and then obviously she advised me of the dangers of smoking while being pregnant and stuff, and she referred me to the smoking [advisor] … She [the midwife] said to me, why don’t you speak to the smoking woman and if you don’t want to do it, you don’t want to do it. But it is worth speaking to her. And I am glad I did, because I had it in my head that I was going to pack in, but I didn’t have a date or anything. But then when I did speak to the smoking woman, I done it [set a quit date] the next day.*

Pregnant woman (PW547), Interview 1

The RPT offered an opportunity for family inclusion within the stop smoking pathway, as partners/relatives were often present for the dating scan. Sometimes this led to results that benefited women and their wider families.

*PW: … the second time [woman received stop smoking information e.g. at RPT] my partner was with me and so they were like showing him as well, why he needs to quit if he is going to be around me. Because it is not good for me, passive smoking and so on. I got it [stop smoking information] twice.*

Interviewer: *And what effect did that have?*

*PW: Well, he [husband] packed in [stopped smoking], so … .*

Pregnant woman (PW547), Interview 1

Maintaining personal autonomy within the decision-making process was essential for the experience of benefit.

*I tried to do it with [first pregnancy] but just didn’t really do it. Like me head wasn’t in it to do it, do you know what I mean? So but this time I was like, right, I am set, I am doing it this time, so ...*

Pregnant woman (PW547), Interview 1

### Follow up systems

There was a variety of types and settings for on-going support with quit attempts. Women reflected on what they wanted: convenient, accessible, reliable services with high levels of support, especially in the early weeks of a quit attempt. Home visits, usually by care assistants, were popular. However, some women preferred attending SSS clinics and found them acceptable, although they did not receive such close support which, sometimes, they missed. Pharmacies were generally seen as a venue to pick up nicotine replacement therapy, and some women developed closer links here too. Features of pharmacies that were important to women included a caring attitude from staff, ease of access to SSS, mid–week support and flexible systems. Women chose the option they preferred within what was available, so unsurprisingly, for the most part, they reported favourably on their follow-up method.

*Oh, it’s been dead good. The midwife [care assistant] that I have been seeing, the one who comes out to me about my smoking, has given me her number. She’s said that I can text her anytime that I feel like I need a tab [cigarette] or anything and she’ll like help us. She comes every week and she’s really nice.*

Pregnant woman (PW715), Interview 1

Where some of these factors (convenience, accessibility, reliability) were lacking, they tended to discourage acceptability. Poor flexibility, a lack of monitoring and feedback, a loss of support beyond 12 weeks and a failure to deliver ongoing encouragement following efforts towards a quit, were all seen as damaging to continuing success at the quit attempt.

*… when I missed the appointment I had no contact with any of them to say, I can’t make it, can I go to a different clinic, even like couldn’t I have made it on the Tuesday? But I had none of that, so I was kind of stuck in a boat where I thought, well, they are not kind of bothered and so I am not bothered.*

Pregnant woman (PW727), Interview 2

## Discussion

Reflecting on women talking about their lives, and visiting them in their homes and communities, it was clear that many of the issues that make it harder to quit were present. The stories they told themselves to allow smoking to continue – or indeed forbid it – were intriguing. In our analysis we found that pregnant smokers, though often taken aback at first by different aspects of the babyClear© intervention (e.g. some intrusive aspects related to the CO monitoring and the risk prevention tool), they also found it supportive and ultimately acceptable. BabyClear© is designed to facilitate the implementation of NICE guidance [14], derived from systematic reviews of previous evidence (including RCTs) and recommends universal screening using CO monitoring, opt out referral to SSS and an enhanced programme of follow-up support. The intervention was designed in response to maternity and stop smoking services finding these initiatives challenging to implement. The reservations of midwives, expressed to Beenstock et al. [15], go some way towards explaining these challenges. Fears of damaging the midwives’ professional relationship with pregnant women by adopting a more aggressive smoking cessation message were commonly voiced [17]. Fear appeal and future punishment have been identified as ways to influence health behaviour [25]; however Peters et al. [26] caution that this approach should only be used when self-efficacy or support for change is high or available, otherwise it could promote health-defeating behaviour instead. This study found that concerns around using the RPT were not manifested by the women we interviewed who received it, and progress was made to normalize the intervention.

The sheer difficulty of delivering system-wide change across organisations was also seen as challenging, but, in spite of these issues, when the resources were made available, the intervention was found to be effective and cost-effective [16], and women’s relationship with maternity staff did not seem to have been damaged, as initially feared by midwives when considering the implementation of NICE guidelines [14]. Indeed, there were specific benefits, including increasing opportunities for other family members to receive the stop smoking message, both in their homes and specifically during delivery of the RPT. Flemming et al. [8, 11, 27], in their systematic reviews, clearly demonstrate the significance of family smoking behaviours upon the pregnant woman, and how, without family support, especially that of partners, women were far less likely to quit smoking themselves.

These findings are supported by evidence for the effectiveness of Behaviour Change Techniques (BCTs), originally identified in the TDF, such as feedback and monitoring, social support, shaping knowledge, natural consequences, reward and threat [25]. Maternity and Stop Smoking Service systems are required which consistently enable the BCTs within the intervention to be delivered. The caveat to this general conclusion relates to the importance of context and circumstances. The way the stop smoking message is communicated by staff (motivational and caring), the systems and resources that back the pathway up, and the flexibility and range of follow-up options that are available to women in a specific location or Trust were all key factors in securing beneficial outcomes.

Despite babyClear© being a standardised package of measures, contexts into which it was implemented varied widely, as did individual women’s experiences [16, 17]. Context is becoming recognised as fundamental to how an intervention is implemented [28, 29], as seen in the realist approach of Pawson and Tilley [30] and the sociological foundations of NPT [21]. Context is being given more prominence, e.g. it forms the overarching element in the Medical Research Council guidance for process evaluations, published in 2014 by Moore et al. [29], now followed by updated guidance for complex interventions by Craig et al. in 2019 [28], where it is also prominent.

We used NPT as a theoretical lens through which to consider the potential for normalization of the babyClear© intervention from a patient perspective. NPT concepts proved helpful in framing the women’s experiences and gave structure to the thematic analysis. The intervention was broadly found to be acceptable to women, in that it made sense to them (coherence) and they accepted it (cognitive participation) and, with support, began to do something about their smoking behaviour (collective action) but women in some contexts reported that the intervention was operating more optimally than in others, leading to variation in acceptability overall (reflexive monitoring).

Within the NPT literature, it is unusual for the theory to be used with patients or individuals; principally, practitioners have been considered, most commonly in HCP teams [31]. However there are exceptions [32–38]. McNaughton [38] has based her doctoral thesis at the individual, patient and HCP level. She found that NPT assisted in identifying the social influences and HCP attributes that affected patient responses to the offer of NHS Health Checks [38]. Anku et al. [39] similarly included patients, as well as practitioners and providers, in their study of combining tuberculosis and HIV services in Ghana. Understanding both the patient perspective and the environment at every level is critical in understanding the overall normalizing of an intervention [21, 40, 41]. The use of NPT to elicit patient perspectives and their influence on normalization is ripe for further study.

### Strengths and limitations

A key strength of this study is that we were able to gain the views, of a population that is considered ‘hard to reach’, on the acceptability of a new intervention. Indeed, one of the researchers (SJ) had previously attended the scan clinic to recruit participants, but this was unsuccessful, in part because women were not willing to extend their time in the clinic to participate in the study. However, data saturation was not reached, so all themes may not be represented. This study was also limited by recruitment methods that resulted in a self-selecting sample of smokers; participants thus may have been unrepresentative, in that women who steadfastly refused to engage with the babyClear© intervention, or who engaged, but saw themselves failing to maintain a quit attempt, may not have volunteered to be interviewed. The study was carried out in only four of the NHS Trust areas participating in the evaluation, because of intervention implementation delays. The fact that these Trusts were able to implement the intervention more readily, though they were located in the same region, may reflect organisational differences between areas, which may mean that the participants we interviewed were more likely to view the intervention as acceptable, than pregnant smokers from the Trusts we were unable to access.

Although we found high levels of acceptability of the intervention, it is unclear how persistent the effects of babyClear© will be postpartum. The focus of this study was smoking during pregnancy and women were not followed up for a significant length of time postpartum. However, in trials which have followed women postpartum, effects of interventions were often no longer significant [19]. In short, whilst many women can be supported to stop smoking for the interlude of their pregnancy, they often resume smoking after this period. Further work is needed to follow-up identified smokers postpartum to explore the long-term impacts of this intervention, including issues relating to relapse.

## Conclusions

The data revealed how the intervention package, and in particular the demonstration of the impact of smoking on the fetus, was communicated using a hard hitting approach, but nevertheless it was generally acceptable to the mothers interviewed. This is an important finding, alongside the outcome study findings which concluded that the babyClear© package, even without the RPT, was effective and cost-effective on a number of measures. There were several crucial aspects in the pathway that were designed to counter some of the cultural forces which make quitting so hard for pregnant smokers – including universal CO monitoring to identify smokers, opt out referral, a caring discourse, follow-up care with high levels of support, flexibility of stop smoking provision and inclusion of family members, all of which were important for success.

Smoking in pregnancy remains a significant cause of avoidable adverse health outcomes for mothers and babies globally, and this paper provides important new evidence to support effective interventions. This intervention was designed to counter some of the barriers, which research has shown, are undermining women’s attempts to quit successfully during pregnancy. The babyClear© package appears to offer significant levels of support and help to women in achieving cessation and is highly acceptable.

## Supplementary information

**Additional file 1: Supplementary information 1.**. Semi-structured interview schedule.

## Data Availability

The datasets generated and/or analysed during the current study are not publicly available due to ethical approval for the study being on the basis of the research team only having access to the raw data but anonymised data are available from the corresponding author on reasonable request.

## Abbreviations

BCT: Behaviour Change Technique
CO: Carbon monoxide
EDD: Expected Date of Delivery
IPiP: Improving Performance in Practice
NHS: National Health Service
NICE: National Institute for Health & Care Excellence
NPT: Normalization Process Theory
RPT: Risk Perception Tool
SSS: Stop Smoking Service
TCCC: Tobacco Control Collaborating Centre
TDF: Theoretical Domains Framework
