# Supplementary material for: Acceptability of a cessation intervention for pregnant smokers: a qualitative study guided by Normalization Process Theory
Source: BMC Public Health. 2020 Oct 6;20:1512. doi: 10.1186/s12889-020-09608-2 (PMC7542132; doi:10.1186/s12889-020-09608-2)
Supplement: Supplementary file 1 — Additional file 1: Supplementary information 1.. Semi-structured interview schedule. [file 12889_2020_9608_MOESM1_ESM.docx]

**Semi-structured interview schedule**

**PT Number:**

**EDD:**

**Today’s date: Weeks gestation:**

| **Themes** |  |
| --- | --- |
| Carbon Monoxide Monitoring | - Have you had your carbon monoxide (CO) level measured? - What did you think about being asked to blow into the CO monitor?   Prompt: How regularly has your CO level been monitored? Is it useful to you? If no, is there any particular reason why?   - Did the midwife say what you expected her to say about your smoking after using the CO monitor? - Did this prompt you to change your smoking behaviour? e.g. stop on your own. |
| Behaviour Change | - Has your smoking pattern changed over the last 4 weeks?   Prompt: Has your pregnancy made you consider your smoking habits?  Prompt: Has your pregnancy affected your smoking? e.g. sickness, increased need.   - Does being pregnant change anything about smoking for you?   Prompt: What have the attitude of other people been towards you?   - Did you make a conscious choice to either smoke/ or not smoke in pregnancy? - Now you are several months on, do you think about smoking differently? - Is there a clash in your mind between smoking and being pregnant? - How much do you actively think about stopping smoking? - If still smoking: Do you think you could stop smoking if you wanted to?   Prompt: Have you tried to quit in the past / previous pregnancies?   - If quit: Do you think you will not smoke again/could give up for good?   Prompt: What about remaining quit after the baby is born?   - Have you taken any steps to stop smoking?   Prompt: Have you contacted the SSS?   - If she has taken steps to stop smoking: Has the midwife/stop smoking advisor helped you to stop smoking?   Prompt: In what ways have the midwife/stop smoking advisor helped or not helped?  Prompt: What prompted you to take a step towards quitting?   - How do you remember to do the things that keep up your quit attempt? - If she has not taken any steps to stop smoking: What are the reasons for not contacting the SSS?   Prompt: Do you feel ready to quit? What prompted you not to take a step towards quitting? Prompt: Do you think your age affects your attitude?   - If still smoking: What do you think will happen if you stop smoking? - If recently quit: What do you think will happen now you have quit? What do you think will happen if you start smoking again? |
| Stop Smoking Services | - If attended SSS: Did you receive advice about identifying goals and how to reach them when quitting? Do you have other long-term goals you are working towards? - If did not attend SSS at all or straight away: How did you feel when it was clear that your non-attendance was being followed up? - For those who have attended the SSS: Are there elements of the service that are helping / hindering you to quit? - Is there anything else you would like from the SSS?   Prompt: e.g. a pregnant women’s clinic, buddy system, support group?   - Do you feel the midwives / SSS staff are working with you on helping you to stop smoking?   Prompt: How well supported (family, friends, healthcare staff) do you feel in stopping smoking?  Prompt: How did you feel about being challenged on your smoking habit by the midwife? (Brief intervention) |
| Relationships with Midwife | - Do you feel the midwives understand what stopping smoking means for you?   Prompt: What was the attitude of the midwife towards you? Prompt: How does stopping smoking fit into your life?   - Has being advised against smoking put you off seeing the midwife?   Prompt: Do you feel your smoking has anything to do with the midwife?   - Why are the midwives / SSS staff so keen for you to stop smoking? - What was your reaction when the midwife showed you the baby doll and the picture of the baby on the screen?   Prompt: Did it make the baby seem more real and how smoking was actually affecting your baby? If so, how did that affect your thinking about your smoking?  Prompt: Are there any other ways that information could be given that would help you understand the relative risk you are taking? |
| Personal views/attitudes | - Are there other things you want to do that might be affected by stopping smoking?   Prompt: positively affected e.g. feel fitter, have more money; or negatively affected e.g. lose friends, sacrifice pleasure.   - Why may you decide not to stop smoking?   Prompt: too hard, like smoking. What about e-cigarettes?   - Could you think about an occasion when you relapsed? - Have either of these (CO monitor or risk perception) shown you any benefits in stopping smoking?   Prompt: If still smoking - What are the benefits to you of continuing to smoke?   - Do you feel more motivated to stop / continue smoking after the advice / RPT? - Have you seen any other health promotion e.g. on TV etc (looking at other contextual factors)? - Did seeing what happens to the baby when you smoke and having a detailed explanation make any difference to how you viewed your smoking? - Are there any issues (constraints) that prevent you from stopping smoking?   Prompt: friends, family, addiction.   - Do you feel you have the necessary support/help available to stop smoking? And to maintain your quit? |
